# Predicting incident cardiovascular disease among African-American adults: A deep learning approach to evaluate social determinants of health in the Jackson heart study

**DOI:** 10.1371/journal.pone.0294050

**Published:** 2023-11-10

**Authors:** Matthew C. Morris, Hamidreza Moradi, Maryam Aslani, Mario Sims, David Schlundt, Chrystyna D. Kouros, Burel Goodin, Crystal Lim, Kerry Kinney

**Affiliations:** 1 Department of Anesthesiology, Vanderbilt University Medical Center, Nashville, Tennessee, United States of America; 2 Department of Psychiatry and Human Behavior, University of Mississippi Medical Center, Jackson, Mississippi, United States of America; 3 Department of Data Science, University of Mississippi Medical Center, Jackson, Mississippi, United States of America; 4 Department of Computer Science, University of North Carolina Agricultural and Technical State University, Greensboro, North Carolina, United States of America; 5 Department of Data Analytics, University of North Texas, Denton, Texas, United States of America; 6 Department of Social Medicine, Population, and Public Health, University of California, Riverside, California, United States of America; 7 Department of Psychology, Vanderbilt University, Nashville, Tennessee, United States of America; 8 Department of Psychology, Southern Methodist University, Dallas, Texas, United States of America; 9 Department of Psychology, University of Alabama at Birmingham, Birmingham, Alabama, Texas, United States of America; 10 Department of Anesthesiology, Washington University in St. Louis, St. Louis, Missouri, United States of America; 11 Department of Health Psychology, University of Missouri, Columbia, Missouri, Texas, United States of America; Luxembourg Institute of Health, LUXEMBOURG

## Abstract

The present study sought to leverage machine learning approaches to determine whether social determinants of health improve prediction of incident cardiovascular disease (CVD). Participants in the Jackson Heart study with no history of CVD at baseline were followed over a 10-year period to determine first CVD events (i.e., coronary heart disease, stroke, heart failure). Three modeling algorithms (i.e., Deep Neural Network, Random Survival Forest, Penalized Cox Proportional Hazards) were used to evaluate three feature sets (i.e., demographics and standard/biobehavioral CVD risk factors [FS1], FS1 combined with psychosocial and socioeconomic CVD risk factors [FS2], and FS2 combined with environmental features [FS3]) as predictors of 10-year CVD risk. Contrary to hypothesis, overall predictive accuracy did not improve when adding social determinants of health. However, social determinants of health comprised eight of the top 15 predictors of first CVD events. The social determinates of health indicators included four socioeconomic factors (insurance status and types), one psychosocial factor (discrimination burden), and three environmental factors (density of outdoor physical activity resources, including instructional and water activities; modified retail food environment index excluding alcohol; and favorable food stores). Findings suggest that whereas understanding biological determinants may identify who is currently at risk for developing CVD and in need of secondary prevention, understanding upstream social determinants of CVD risk could guide primary prevention efforts by identifying where and how policy and community-level interventions could be targeted to facilitate changes in individual health behaviors.

## Introduction

Cardiovascular disease (CVD), which includes fatal and nonfatal coronary heart disease (CHD), myocardial infarction (MI), and stroke, is the leading cause of death in the United States, accounting for 1 in 4 deaths [1,2]. Staggering racial disparities exist in CVD morbidity and mortality, with Non-Hispanic Black (NHB) adults exhibiting the highest CVD mortality across all ages compared to other racial and ethnic groups [3]. In 2017, CHD death rates (per 100,000) for adults ages 35 and older were 204 and 182 for NHB and Non-Hispanic White (NHW) adults, respectively [4]. Overall, mortality rates for NHB as compared to NHW adults are estimated to be 30% higher for CHD and 45% higher for stroke [5].

There is now strong empirical support for the association between social determinants of health (SDOH) and CVD risk [6]. Social determinants refer to the environments in which people are born, live, and age, and include socioeconomic status (SES), social support, neighborhood and housing conditions, exposure to stressors and discrimination, and access to quality education, food, and health care. Socioeconomic and environmental risk factors for CVD lie upstream relative to more downstream, individual-level behavioral and biological risk factors. Increased risk for CVD morbidity and mortality has been linked to a host of social determinants, including lower per capita household income [7], higher rates of neighborhood poverty, higher levels of neighborhood violence and crime exposure [8], unemployment, greater percentages of single family households, overcrowding, greater racial segregation [8], lower levels of perceived social support [9], reduced access to medical care [6,8,10,11], limited neighborhood walkability and access to public open spaces [12], and the presence of food deserts [13]. The added value of social determinants as predictors of CVD above and beyond traditional biobehavioral risk factors (e.g., smoking, systolic/diastolic blood pressure, eating behaviors) has yet to be determined.

Psychosocial factors such as stress and discrimination are also considered social determinants of risk for CVD and typically lie downstream relative to socioeconomic and environmental factors. Adults who report experiencing four or more early life stressors are 2.2 times more likely to develop CHD and 2.4 times more likely to develop stroke [14]. Meta-analytic findings also suggest a medium effect size for the association between early adversity and CVD [15]. Stressors, such as discrimination and being the victim of an assault, are associated with increased risk for CVD (e.g., elevated blood pressure, atherosclerosis) and mortality [16–20]. Conversely, growing up in an environment with lower stress levels and higher SES is associated with lower CVD risk in adulthood [21]. According to minority stress theory [22], racial disparities in CVD are driven, in part, by disproportionately high exposure to stressors across the lifespan [6,23–28]. Most cross-sectional studies find higher rates of stressful events in NHB compared to NHW adults, though few carefully control for SES [29]. Moreover, relations between discrimination and CVD appear stronger for NHB than non-minority adults [16,30]. In the Jackson Heart Study (JHS), greater risk for CVD has been associated with higher negative affect (i.e., depressive symptoms, cynicism, anger) and stress levels (i.e., weekly stress levels, major life events) [31–36].

Racial disparities in CVD are complex and multi-faceted [37], yet studies have primarily examined risk and protective factors separately. One significant barrier to this research is that traditional analytic approaches do not include the wide array of cross-domain (e.g., environment, behavior, biology) and cross-level (geographic, socioeconomic, interpersonal, individual) exposures implicated in CVD risk, which exert statistically significant but weak individual effects. Data-driven techniques based on machine learning (ML) are well-suited for CVD risk prediction because they can overcome the restrictive modeling assumptions and limitations on number of predictors that characterize traditional multivariable regression approaches. Despite the promise of SDOH for improving CVD risk prediction beyond traditional risk scores (e.g., Framingham) that ignore SES [38], few studies have determined whether these predictors add predictive value to standard/biobehavioral CVD risk factors. One study using ML showed that greater area-level social service resources (i.e., food, employment, and nutrition) were associated with lower CVD risk (i.e., lower body mass index [BMI]) [39]. The present study addressed key gaps in the extant research by adopting ML to evaluate the predictive performance of social determinants for first CVD events among NHB adults in the JHS across multiple levels of their social ecology; these predictors included experiences of discrimination, low SES, neighborhood violence, and nearby physical activity facilities [26,40–44]. We hypothesized that models including social determinants (i.e., psychosocial, socioeconomic, and environmental factors) would exhibit superior predictive accuracy for incident CVD as compared to models including only standard/biobehavioral CVD risk factors.

## Materials and methods

### Participants

The JHS is a longitudinal cohort study focused on understanding the emergence of CVD [45]. During the baseline assessment, which occurred between March 2000 and September 2004, NHB adults, ages 21 to 94, were recruited from the tri-county area (Hinds, Madison, and Rankin) of the Jackson, Mississippi metropolitan area. Participants were excluded if they had a history of CVD at baseline as evident in any of the following conditions: self-reported history of MI; self-reported history of cardiac procedure; self-reported history of physician-diagnosed stroke; history of CHD from electrocardiogram and self-report; and self-reported history of carotid angioplasty. All JHS participants provided written informed consent and JHS was approved by the Institutional Review Boards of The University of Mississippi Medical Center, Jackson State University, and Tougaloo College.

#### CVD outcomes

CVD events, which included CHD (i.e., definite or probable MI, definite fatal CHD, cardiac procedures), stroke (definite or probable), and heart failure (HF; JHS surveillance and event adjudication started on January 1, 2005), were carefully documented and verified through data linkage hospital discharge lists and National Death Index and review of medical records of CVD-related hospitalizations and death certificates to adjudicate CVD events and deaths [46]. First CVD events were determined over a 10-year follow-up period.

### CVD risk factors

#### Standard/Biobehavioral risk factors

Standard/biobehavioral CVD risk factors assessed at baseline included BMI, systolic and diastolic blood pressure, ankle brachial index, blood pressure medication status, total cholesterol, low density lipoprotein (LDL) cholesterol, high density lipoprotein (HDL) cholesterol, triglycerides, fasting glucose, hemoglobin A1C (HbA1c), alcohol drinking, smoking, physical activity, diet, age, sex, and waist circumference. BMI was determined as weight (kilograms) divided by height (meters squared). Hypertension status was derived from the Joint National Committee on Prevention (JNC-7) and defined as systolic blood pressure ≥ 140, diastolic blood pressure ≥ 90, or use of antihypertensive medications [47]. Diabetes status was derived from the American Diabetes Association (ADA) criteria and defined as fasting glucose ≥ 126 mg/dL, HbA1c ≥ 6.5%, or use of diabetic medication within 2 weeks of clinic visit [48]. Cholesterol measures included fasting LDL level (mg/dL), fasting HDL level (mg/dL), and total fasting cholesterol level (mg/dL). Additional CVD biospecimens included fasting triglyceride level (mg/dL), HbA1c (National Glycohemoglobin Standardization Program units [%]), and fasting plasma glucose level (mg/dL). Medications (anti-hypertensive, anti-diabetic) were determined by self-report. Alcohol use was assessed by self-report (i.e., frequency of use in the past 12 months). Smoking was assessed by self-report tobacco use forms (27-items) and used to determine current and history of cigarette smoking; prior work suggests CVD risk remains elevated in former- smokers for 3–15 years compared to those with no history of smoking [49,50]. Physical activity was determined by the Physical Activity (PA) scale from the Active Living Index (30-item [51]), and computed as a categorical variable (0 = *poor health* (0 min/week of physical activity); 1 = *intermediate health* (1–49 min/week of moderate activity or 1–74 min/week of vigorous activity or 1–149 min/week or moderate+vigorous activity); 2 = *ideal health* (≥150 min/week of moderate activity or ≥75 min/week of vigorous activity or ≥150 min/week of moderate+vigorous activity). Diet was determined by the 158-item Food Frequency Questionnaire (FFQ). The following components of a 2000-kcal diet were used to determine nutrition categories (i.e., *poor health* = 0–1 components; *intermediate health* = 2–3 components; *ideal health* = 4–5 components) based on American Heart Association guidelines [52]: (1) ≥ 4.5 cups/day of fruits and vegetables; (2) > 3.5 ounces twice/week of fish; (3) < 1500 mg/day of sodium; (4) < 450 kcal/week of sugary beverages; (5) ≥ 3 servings/day of whole grains).

#### Social determinants: Psychosocial factors

Psychosocial factors assessed at baseline included perceived daily discrimination, lifetime discrimination, burden of lifetime discrimination, perceived depressive symptoms, and perceived stress levels. Daily discrimination was assessed with a 9-item measure based on the scale developed by Williams and colleagues [53] (good internal consistency: alpha = 0.88) [54]. Lifetime discrimination was determined through a self-report measure assessing the occurrence of unfair treatment across 9 domains (adequate internal consistency: alpha = 0.78) [31,54]. Burden of lifetime discrimination (i.e., interference related to discrimination) was assessed by a measure exhibiting adequate internal consistency (alpha = 0.63). Depressive symptoms were determined by the 20-item [55] version of the Center for Epidemiological Studies Depression Scale (CES-D), with higher scores reflecting greater depression severity in the prior week (alpha = 0.82). Perceived stress was determined by the Global Perceived Stress Scale (GPSS; 8 items), adapted for the JHS from other validated stress measures [56] with adequate psychometric properties (alpha = 0.72) [34]. Higher scores reflected greater perceived stress levels over a 12-month period across multiple domains (e.g., employment, relationships, neighborhood, basic needs). In addition, the Weekly Stress Inventory (WSI; 87 items [57]) was used to capture minor stressors (e.g., work tasks, finances, household tasks, relationships) experienced by participants. Higher WSI-impact scores reflected greater stress ratings for events occurring in the past week. The WSI has excellent psychometric properties (alpha = 0.98) [34].

#### Social determinants: Socioeconomic factors

Socioeconomic factors assessed at baseline included family income (categories: less than $5,000; $5,000–7,999; $8,000–11,999; $12,000–15,999; $16,000–19,999; $20,000–24,999; $25,000–34,999; $35,000–49,999; $50,000–74,999; $75,000–99,999; $100,000 or more), occupation (U.S. Department of Labor Standard Occupational Classifications: management/professional, service, sales, farming, construction, production, military, sick, unemployed, homemaker, retired, student, other), education (0 = less than high school; 1 = high school graduate/GED; 2 = attended vocational school, trade school, or college), and insurance status (any insurance; insurance type [uninsured, public only, private only, private & public).

#### Social determinants: Environmental factors

A complete list of the environmental factors (census tract-level indicators) that comprise SDOH is included in the S1 Table [8,58]. Measures varied according to spatial function (i.e., simple or kernel density) and area (i.e., ½ mile, 1 mile, or 3 mile radius). Environmental data reflecting densities of physical activity resources and “favorable” food stores were obtained from the National Establishment Time-Series (NET-S) and Nieslen/TDLinx Service Supermarket Retail Category databases for the years 2000 to 2010, and linked to JHS baseline data using geocoded participant addresses as described elsewhere [59,60]. Favorable food store density was based on the density of groceries, supermarket chains and non-chain stores, and fruit and vegetable markets. Unfavorable food store density was based on the density of convenience stores, bakeries, candy/nut shops, ice cream stores, liquor stores, alcoholic drinking places, and fast food stores [59]. Physical activity facility density was based on the following resources: biking, bowling, dance, golf, indoor conditioning, physical activity instruction, swimming, team and racquet sports, and water activities. Environmental factors in the present study included Census-derived measures of median household income, percentage living below poverty, percentage black non-Hispanic residents, percentage white non-Hispanic residents, favorable food stores within 3 miles, physical activity facilities within 3 miles, percentage residential land use per square mile, and population density. Self-report measures of environmental characteristics assessed neighborhood problems (i.e., age- and sex-adjusted scale including participant reports of excessive noise, heavy traffic or speeding cars, lack of access to adequate food and/or shopping, lack of parks and playgrounds, trash and litter, and lacking or poorly maintained sidewalks in their neighborhoods), neighborhood social cohesion (i.e., age- and sex-adjusted participant reports of living in a close knit neighborhood, people willing to help neighbors, neighbors generally getting along, neighbors who can be trusted, neighbors who share the same values, neighborhood safety from crime), and neighborhood violence (age- and sex-adjusted scale including items assessing how often participant reported fights with weapons, violent arguments, gang fights, sexual assaults or rapes, and/or robbery or muggings in their neighborhoods).

### Data curation

We used a light-touch approach to participant exclusion and feature imputation to avoid affecting subsequent model interpretations with any prior assumptions. Data manipulation was minimized in the process of training and testing models. Accordingly, only participants with a history of CVD at baseline were excluded. Missing features imputation were conducted with constant values (medians) to preserve the informativeness of each feature in the dataset [61]. ML-based imputation strategies were eschewed due to concerns that learning from the existing correlational structure could affect the determination of feature importance. Preliminary analyses using Python’s Scikit-learn iterative random forest imputer [62] did not improve model accuracy. As a result, all missing values were imputed using the median and no missing features resulted in participant removal from analyses.

### Feature sets

To evaluate the effect of different sets of features on the model’s prediction accuracy, three feature sets were considered. In the first Feature Set (FS1), demographics and standard/biobehavioral CVD risk factors were considered as predictive features; the second Feature Set (FS2) combined psychosocial and socioeconomic features with FS1, and the third Feature Set (FS3) utilized FS2 along with environmental features.

### Modeling algorithms and evaluation

We evaluated three modeling algorithms for predicting 10-year CVD risk among JHS patients. For each algorithm, different feature sets as inputs were used to model the survival function. Comparisons between the models’ estimated risks were conducted using Antolini time-dependent Concordance Index (CI) to account for non-proportional hazard model used in this study [63,64]. Please note that Antolini CI is equivalent to ‘Harrell’s C’ for survival models with proportional hazards [65,66]. HyperOpt [67]–an open-source Bayesian optimization library–was used to address models’ sensitivity to hyper-parameters, increase the models’ accuracy, and to facilitation study replication. Hyper-parameter tuning was performed on random train, validation, and test splits of 60%, 20%, and 20%, respectively. Following hyper-parameter tuning, model evaluation was conducted by 10-fold cross-validation to report the model’s average CI. All implementations were conducted in Python 3.8 using PyTorch 1.10, PyCox 0.2.3, and Scikit-Survival 0.17.2. The following three modeling algorithms were used in the present study:

#### Deep neural network

Neural Network (NN)-based models have been shown to improve prediction accuracy [68]. The present study implemented Deep Neural Network (DNN) models based on DeepHit [65]. DeepHit learns the distribution of survival time directly from the data without any prior assumption(s) about the underlying stochastic process. As a result, predictions depend directly on features in the dataset. The loss function of DeepHit is designed to handle censored data for survival analysis. For DeepHit, the number of layers, number of neurons in each layer, dropout rate, optimization algorithm, learning rate, activation function, and batch size are all considered as hyper-parameters and optimized.

#### Random survival forest (RSF)

As an ensemble of tree-based learners, this algorithm ensures individual trees are de-correlated. Each tree is built on a bootstrap of the original training dataset and split criteria in each node include a random subset of features [69]. Final prediction results comprise the combined predictions from all trained trees. For this algorithm, we used scikit-survival implementation with number of estimators and max depth as hyper-parameters [70].

#### Penalized cox proportional hazards (CPH)

This algorithm was included as a comparison model due to its ease of implementation and low computational requirement. Penalized CPH models were implemented in scikit-survival with regularization parameter and convergence criteria as hyper-parameters.

### Model interpretability

ML models are often viewed as black-box procedures yielding little insight or interpretability except for predictions of outcomes. However, recent improvements have been made in the generation of robust and interpretable insights from complex ML models [71]. Shapley Additive Explanation (SHAP [72]) values have gained attention because they can facilitate interpretation of complex ML models with high accuracy and robustness. By comparing SHAP values generated for input features, it is possible to assess the extent to which changes in the inputs influence the final model’s prediction and, hence, to evaluate feature importance for complex models. The present study evaluated standard/biobehavioral risk factors and social determinants as features in a complex DNN model for CVD risk prediction. After optimizing model hyperparameters, training, and evaluating the model accuracy, the model was subsequently retrained on the full dataset using the same parameters; in this manner, the model could learn all existing interactions in the dataset. As recommended by SHAP best practice guidelines for calculation of the required background samples, we applied the K-Nearest Neighbor clustering algorithm (K = 100) to the dataset; this provided a total of 100 cluster centroids to be used for SHAP value calculation. SHAP values were then generated for input features based on the trained model and the background samples, providing insight into feature importance. Feature importance was then reported as the absolute mean value of the effect on the final model prediction.

## Results

### Study population

During the 10-year follow-up period of NHB adults with no history of CVD at baseline (n = 3,980), 382 participants experienced at least one CVD incident: there were 139 cases of incident CHD, 221 cases of incident heart failure, and 104 cases of incident stroke. Event was defined as the first adjudication of any CVD incident. Descriptive characteristics for NHB adults with and without CVD are presented in Table 1. Participants exhibited a mean age of 53.8 years and 64% were female.

**Table 1:** Descriptive characteristics for patients with and without incident CVD.

Baseline Characteristic	Incident CVD (n = 382)	No Incident CVD (n = 3,598)	All (n = 3,980)	Missing values
Sex				0
Male (%)	154 (40)	1,265 (35)	1,419 (36)	
Female (%)	228 (60)	2,333 (65)	2,561 (64)	
Age (*SD*)	62.6 (11)	52.8 (12)	53.8 (12)	0
Education				12
Less than high school (%)	117 (30)	510 (14)	627 (15)	
Greater than High school (%)	265 (70)	3,076 (86)	3,341 (85)	
Occupation				8
Employed (%)	381 (>99)	3,569 (>99)	3,950 (>99)	
Not Employed (%)	1 (<1)	21 (<1)	22 (<1)	
Smoking status				
Current smoker (%)	61 (16)	395 (11)	456 (11)	36
Ever smoker (%)	149 (39)	1,019 (28)	1,168 (29)	9
Insurance Status				17
Yes (%)	348 (91%)	3,081 (86%)	3429 (86%)	
No (%)	33 (9%)	501 (14%)	534 (14%)	
BMI *(SD)*	31.9 (7)	31.6 (7)	31.6 (7)	7

#### Model accuracy

Penalized CPH, RSF, and DNN models were evaluated using CI with three sets of features as inputs (FS1, FS2, FS3); mean CI of 10-fold cross-validation and corresponding standard deviations for models are presented in Table 2. The DNN model exhibited the highest overall accuracy across feature sets. With higher numbers of input features, the accuracy of the RSF and CPH models decreased. In contrast, the DNN model exhibited consistent performance regardless of the number of input features, and detected the best set of features predictive of the outcome of interest in a high dimensional space.

**Table 2:** Concordance indices for predictive models.

Feature sets	Deep Neural Network	Random Survival Forest	Penalized CPH Regression
	Mean (*SD*)	Mean (*SD*)	Mean (*SD*)
Standard risk factors (FS1)	0.76 (0.03)	0.75 (0.07)	0.74 (0.06)
Psychosocial/socioeconomic + standard risk factors (FS2)	0.76 (0.03)	0.75 (0.06)	0.74 (0.06)
Environmental + Psychosocial/socioeconomic + standard risk factors (FS3)	0.76 (0.03)	0.72 (0.05)	0.72 (0.06)

Note: CPH = Cox proportional hazards.

### Feature importance

**Fig 1:**
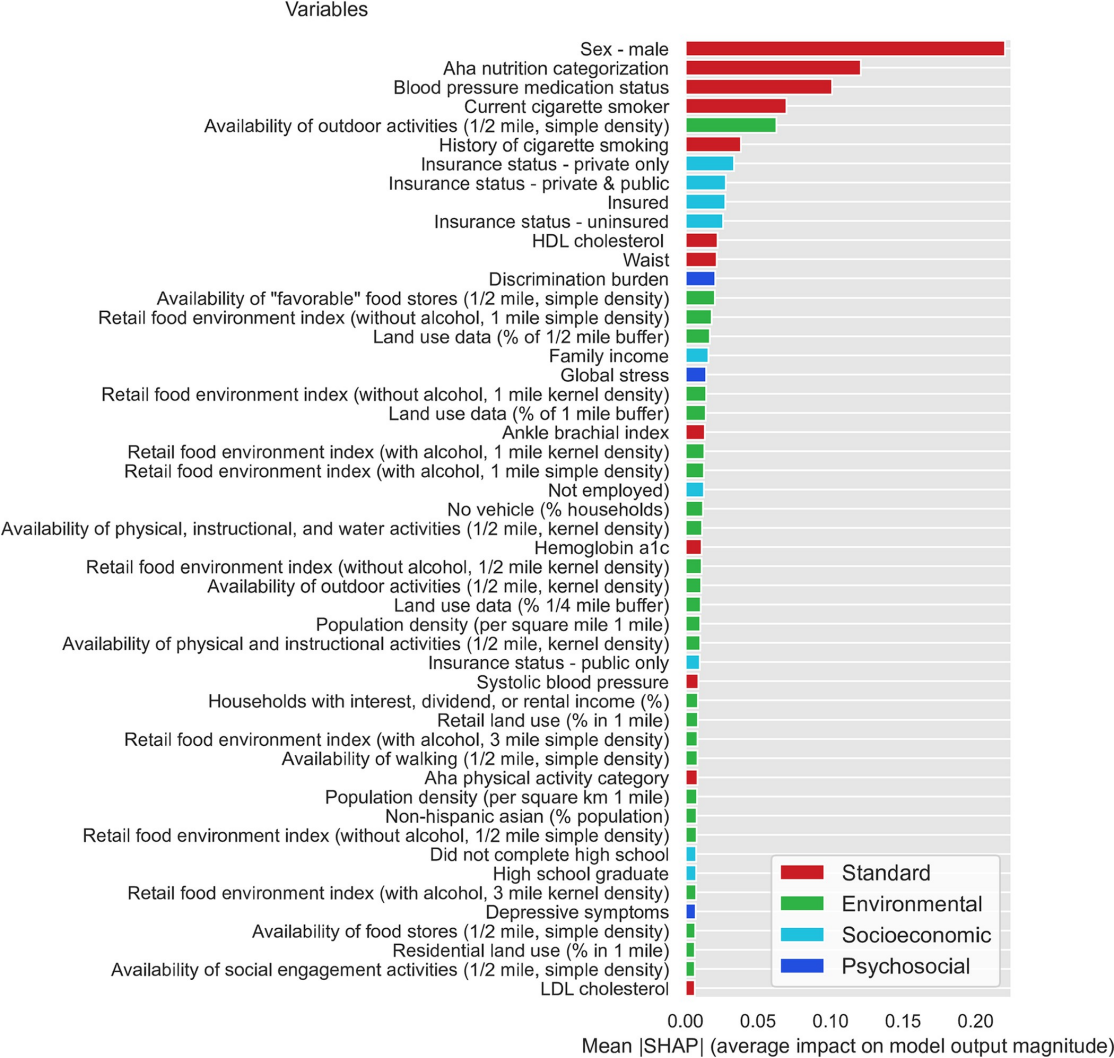
Relative importance for the top 50 study features sorted by mean absolute SHAP value.

**Fig 2:**
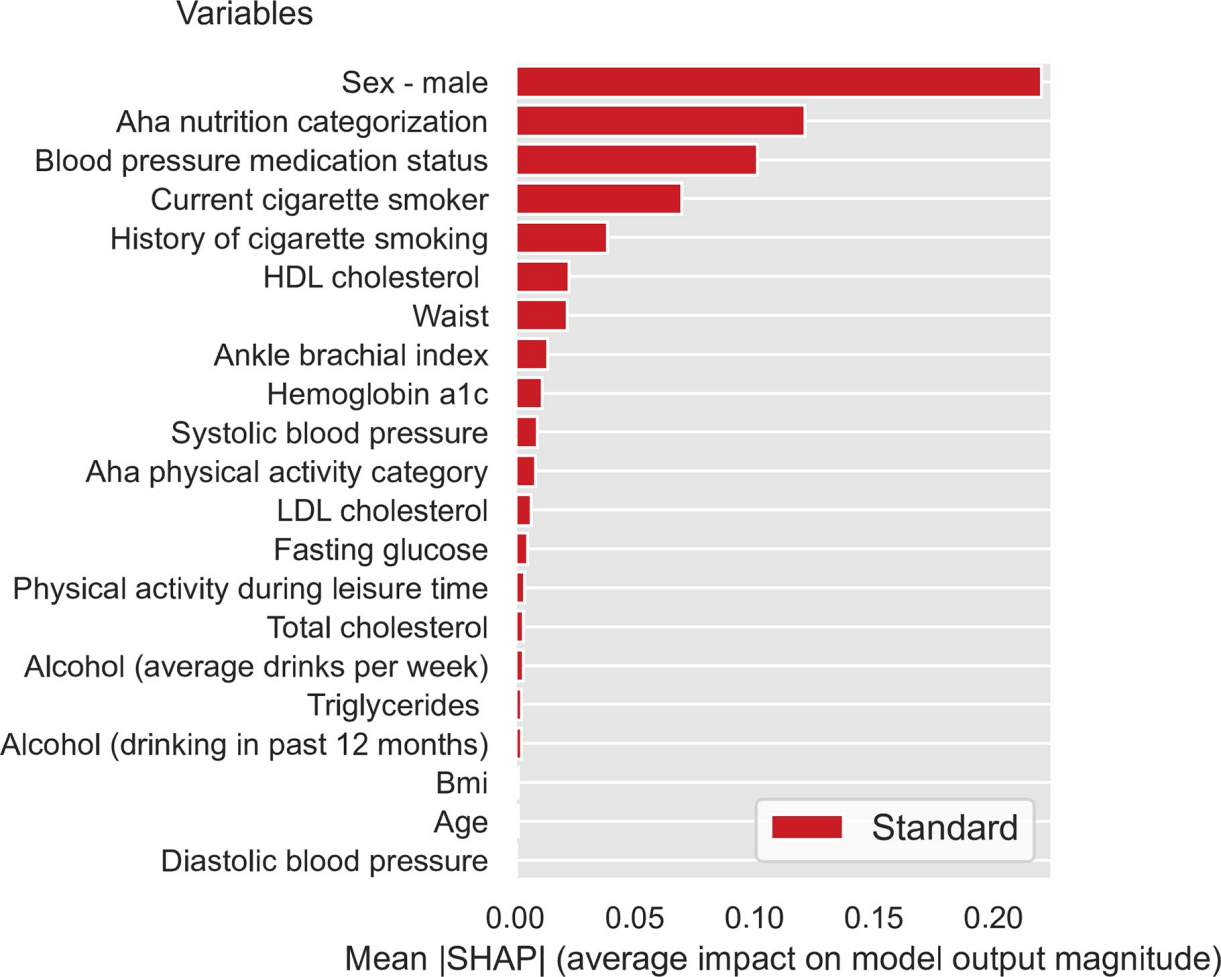
Relative importance for standard CVD risk features sorted by mean absolute SHAP value.

**Fig 3:**
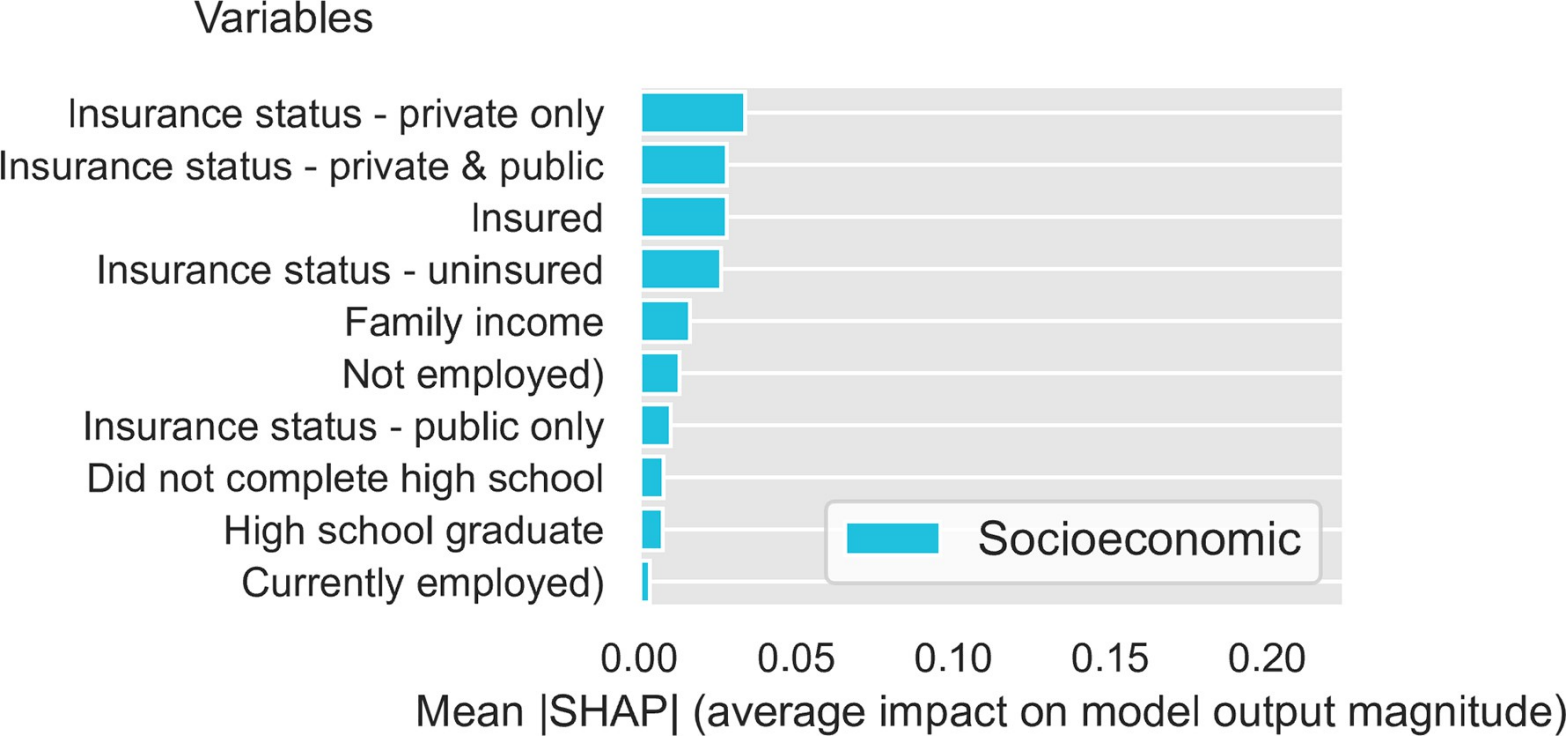
Relative importance for socioeconomic features sorted by mean absolute SHAP value.

**Fig 4:**
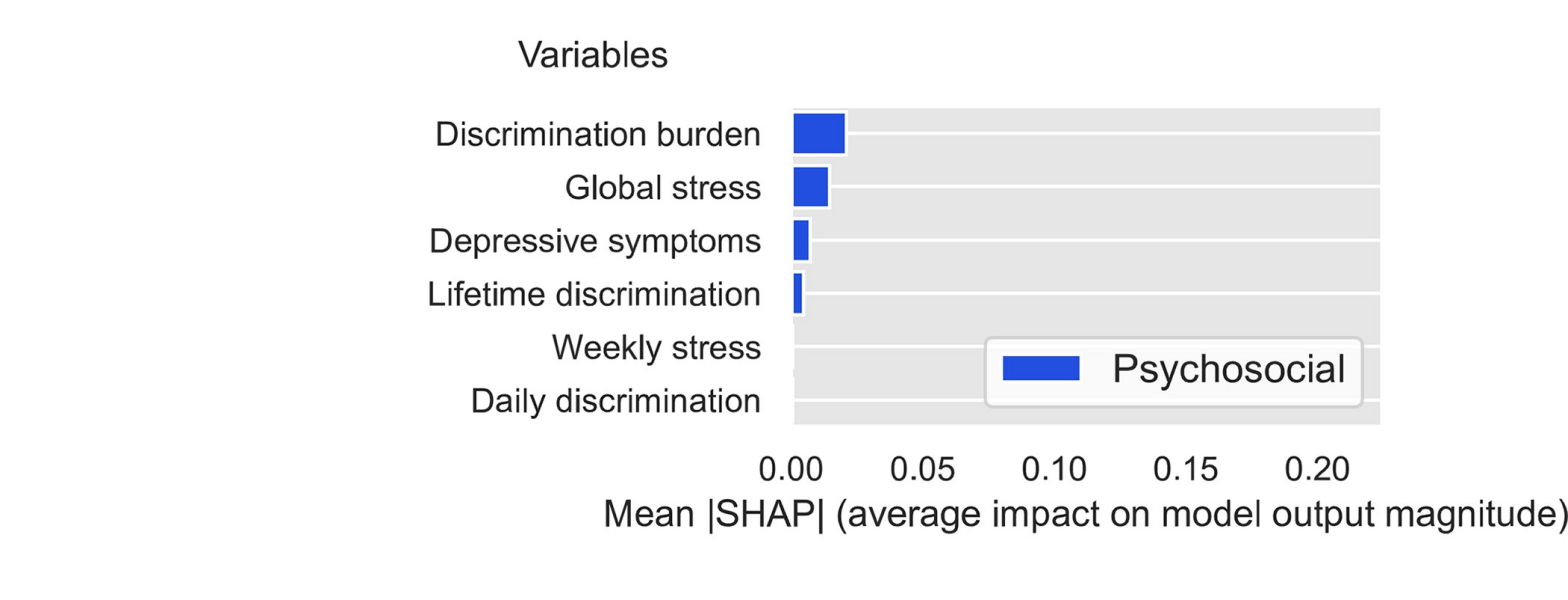
Relative importance for psychosocial features sorted by mean absolute SHAP value.

**Fig 5:**
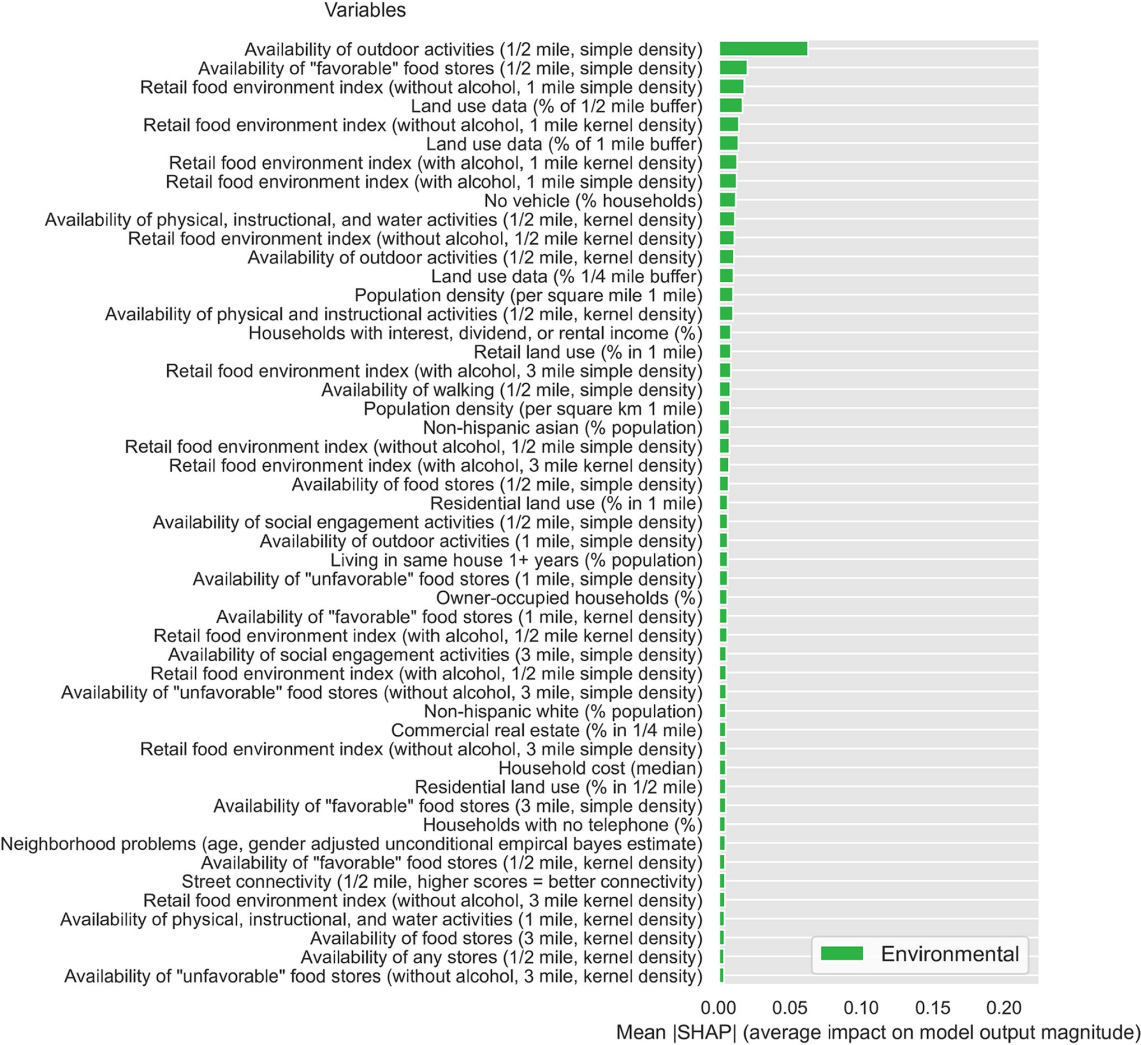
Relative importance for environmental features sorted by mean absolute SHAP value.

To evaluate feature importance, we calculated the SHAP values for all the features in the dataset using the DNN model trained on the FS3 feature set (i.e., all standard/biobehavioral, psychosocial/socioeconomic, and environmental factors). Fig 1 presents the relative importance (average impact on final model output) for the top 50 features investigated in this study, sorted by their absolute mean SHAP value (relative importance for all features is presented in the S1 Table). Seven of the top 15 features–including five of the top 10 –were standard/biobehavioral CVD risk factors, including sex (male), nutrition, blood pressure medication status, cigarette smoking status (current smoker, history of smoking), HDL cholesterol, and waist circumference. Notably, 8 of the top 15 features were SDOH. These included four socioeconomic factors (insurance status and type), one psychosocial factor (discrimination burden), and three environmental factors. The latter factors included area-level composite variables that reflect density of outdoor physical activity resources (including instructional and water activities) and favorable food stores (i.e., grocery stores, supermarket chains and non-chain stores, fruit and vegetable markets). Relative importance is also presented separately for standard CVD risk (Fig 2), socioeconomic (Fig 3), psychosocial (Fig 4), and environmental (Fig 5) features.

## Discussion

Based on prior work, the extent to which SDOH—including psychosocial, socioeconomic, and environmental factors–can improve predictive accuracy for incident CVD beyond standard/biobehavioral risk factors was unclear. To address this gap, the present study used ML models to determine overall predictive performance for incident CVD events (i.e., CHD, stroke, and/or HF) among NHB adults followed over time in the JHS, and to assess the relative importance of standard/biobehavioral and social determinant features in these models. The DNN model provided more accurate predictions regarding incident CVD than RSF or CPH models. Contrary to our hypothesis, overall predictive accuracy for DNN models did not improve when adding SDOH. Whereas accuracy was stable across feature sets for DNN models, decreases in accuracy were observed for RSF and CPH models with higher numbers of inputs. This was likely due to high dimensionality of the input datasets and inability of RSF or CPH models to accurately detect important features required to maintain or increase accuracy. Taken together, these results highlight the promise of DNN over RSF and CPH models for predicting incident CVD, but suggest that the psychosocial, socioeconomic, and environmental factors did not appreciably improve predictive accuracy for first CVD events beyond biobehavioral risk factors.

Recent work demonstrates improved prediction of first fatal or non-fatal CVD events using ML algorithms as compared to conventional statistical approaches focused on standard risk factors or scores typically derived from routinely collected clinical data [73–78]. In addition, ML approaches have shown that neighborhood-level predictors (e.g., prevalence of obesity, rates of binge drinking and leisure-time physical activity) were associated with higher rates of CHD and stroke [79]. Predictive accuracy for DNN in the present study was comparable to another study using Neural Networks to predict first CVD events in 423,604 participants in the UK Biobank (AUC-ROC: 0.755, 95% CI: 0.750–0.760); the latter study included 4,801 incident CVD cases within 5 years of baseline assessment and a host of features that overlapped with the present study (e.g., diet, physical activity, sociodemographics, lipid profile, body composition, depressive symptoms) but notably did not include environmental factors [73]. The following sections explain the reasons the inclusion of psychosocial (e.g., stress levels and depressive symptoms), socioeconomic (e.g., family income and educational attainment), and environmental (e.g., neighborhood poverty) risk factors did not improve overall predictive performance for first CVD events beyond biobehavioral risk factors (e.g., diet, blood pressure, smoking).

First, it is important to note that even though overall predictive accuracy was not improved by adding social determinants as input features, this does not imply that these features are not important predictors of incident CVD. Analyses of feature importance (i.e., Shapley Additive Explanation [SHAP] values) can aid interpretation of complex DNN models and complement overall indicators of predictive accuracy (concordance index [CI]) by providing information on the relative importance of input features. SHAP values showed that standard/biobehavioral risk factors comprised seven of the top 15 predictors of first CVD events, However, the relative importance of psychosocial, socioeconomic, and environmental factors cannot be discounted. Discrimination burden, insurance status, and outdoor physical activity resources and ranked higher in importance than well-established standard/biobehavioral risk factors such as HbA1C, systolic block pressure, physical activity levels, and LDL cholesterol [80].

Second, conceptual models depict pathways linking upstream (e.g., economic stability, neighborhood environment, structural discrimination, and access to education, health care, and healthy food) and midstream (e.g., exposure to stressors, experiences with discrimination, health behaviors, diet) social determinants to downstream (e.g., hypertension, obesity, lipid profiles, HbA1c) risk factors for CVD [81,82]. These models highlight “trickle-down effects” of socio-contextual factors on social position, lived experiences, biobehavioral responses, and, ultimately, CVD development and progression for marginalized groups [82]. One interpretation of the present findings is that this ‘trickle’ takes time: whereas biological determinants tell us who is currently at risk for developing CVD and in need of secondary prevention, social determinants tell us who may be at risk for developing CVD and could benefit from primary prevention. If social determinants exert their influence through biological determinants, then they may be less useful for forecasting incident CVD and more useful as targets for preventive policy, community, and individual interventions.

Third, a priori distinctions made in the present study between standard/biobehavioral, psychosocial, socioeconomic, and environmental factors may ignore other important dimensions and sources of cross-category overlap. For example, social determinants are likely to differ according to their timing and duration, with features such as education and experiences with discrimination exerting a cumulative effect on CVD risk over a lifetime as compared to more proximal features (e.g., stress levels, current neighborhood violence, depressive symptoms) that influence CVD risk factors on a day-to-day basis. It is also unclear whether and to what extent correlations among input features can influence accuracy and measures of feature importance in Neural Network models. Standard/biobehavioral factors such as physical activity and diet are likely to be correlated with environmental features such as walkability and density of favorable food stores, respectively. In addition, the environmental factors included identical resource measures that differed only by spatial function (i.e., simple or kernel density) and/or area (i.e., ½ mile, 1 mile, or 3 mile radius) as well as resource measures that differed only slightly by content (e.g., food stores with and without alcohol). To our knowledge, potential problems with multicollinearity in Neural Network models–including the extent to which correlations among inputs influence non-linear activation functions and advanced regularization methods—have yet to be evaluated. Notably, strong correlations among similar variables in the present study (e.g., current smoker and history of smoking) did not prevent them from emerging simultaneously as important features in SHAP analyses.

Fourth, it should be noted that DNN models typically require large datasets to be trained effectively. Therefore, given the relatively small size of the dataset, the DNN model may not outperform less complex models such as CPH or RSF. This could be a plausible reason why the addition of SDOH did not improve the predictive accuracy of our model, despite being among the top predictors of the first CVD event. Another possible explanation is that SDOH may not have a significant impact on predicting first CVD event beyond the predictive contribution of other risk factors. We hypothesize that this may be due to the complex and multifactorial nature of CVD risk. Finally, it is possible that the attainable accuracy for the provided dataset has been reached. In this regard, we acknowledge that the accuracy we achieved is similar to other studies utilizing full cohort [83].

The present findings have important implications for efforts to prevent the onset of CVD. First, more upstream SDOH are unlikely to improve accuracy of ML models that seek to distinguish NHB adults in terms of their risk for first CVD events over a 10-year follow-up period. While it is possible that assessing the cumulative impact of sociopolitical and economic factors on CVD risk at higher levels of the social ecology would improve predictive accuracy over a longer time frame, our results suggest that careful assessment of SDOH may not yield dividends for more immediate risk stratification over and above standard/biobehavioral measures. Second, our results suggest that identifying connections between upstream (e.g., neighborhood resources for physical activity) and midstream (e.g., stress levels) determinants [81] could prove useful for primary prevention of CVD. Neighborhoods identified as having higher rates of upstream social determinants of CVD risk could be prioritized for delivery of preventive interventions. Neighborhood features identified as important for incident CVD prediction could then be targeted by policy and community-level interventions to facilitate changes in–and remove barriers to—individual health and wellness behaviors.

### Limitations

Limitations of the present study may provide directions for future research. First, as noted above, the degree to which strong correlations among feature inputs influences SHAP values for relative importance remains unclear. Second, the relative importance of features with higher levels of missing data may be underestimated in the present study despite the use of median imputation. The top three features based on missingness among JHS participants were depressive symptoms (n = 1,309), weekly stress levels (n = 1,663), and percent retail land use within a 1/4 mile radius (n = 580). Hence, the importance of these features for predicted CVD risk should be interpreted with caution. Third, the adjudication of HF in the JHS was initiated in 2005 despite enrollment beginning in 2000. Hence, there may have been a small number of participants for whom HF events were not captured and who may have been misclassified. Fourth, focusing primarily on middle-to-older aged adults and including a relatively short (10 year) follow-up period may have favored biobehavioral over other risk factors. Studies following younger individuals over a longer time frame may be better-suited to capturing more gradual effects of SDOH on incident CVD.

## Conclusion

The present findings highlight important features associated with risk for incident CVD across multiple levels of the social ecology and argue in favor of a biopsychosocial approach to CVD risk and prevention. Although SDOH did not augment predictive accuracy for CVD events over a 10-year period, they emerged as important features of predictive models that ranked above even well-established behavioral and biological risk factors. Future studies should leverage ML approaches to feature importance in order to guide prevention efforts by identifying salient points along the stream where an individual’s risk for developing subsequent CVD may be diverted.

## Supporting information

S1 Table Relative importance of all features sorted by absolute mean SHAP values. (DOCX)

## Abbreviations

CVD: cardiovascular disease
CHD: coronary heart disease
MI: myocardial infarction
NHB: Non-Hispanic Black
NHW: Non-Hispanic White
SES: socioeconomic status
JHS: Jackson Heart Study
HF: heart failure
ML: machine learning
DNN: Deep Neural Network
RSF: Random Survival Forest
CPH: Cox Proportional Hazards
SHAP: Shapley Additive Explanation
SDOH: social determinants of health
