# Supplementary material for: Predicting incident cardiovascular disease among African-American adults: A deep learning approach to evaluate social determinants of health in the Jackson heart study
Source: PLoS One. 2023 Nov 10;18(11):e0294050. doi: 10.1371/journal.pone.0294050 (PMC10637695; doi:10.1371/journal.pone.0294050)
Supplement: S1 Table — (DOCX) [file pone.0294050.s001.docx]

**S1 Table**: **Relative importance of all features sorted by absolute mean SHAP values**

| **Feature** | **Format** | **Rank** | **Mean \|SHAP\|** | **Feature Category** |
| --- | --- | --- | --- | --- |
| Sex - Male (sex) | 0 = female; 1= male | 1 | 0.219952225 | Standard |
| Nutrition Categorization (nutrition3cat) | 0 = poor health;  1 = intermediate health;  2 = ideal health | 2 | 0.120787952 | Standard |
| Blood pressure medication status (bpmeds) | 0 = no; 1 = yes | 3 | 0.101076458 | Standard |
| Cigarette Smoking Status (currentsmoker) | 0 = no; 1 = yes | 4 | 0.069433025 | Standard |
| 1/2 mile simple outdoor total physical activities + instructional + water (s0opai) | Higher scores = more activity resources available within area to resident | 5 | 0.062604695 | Environmental |
| History of Cigarette Smoking (eversmoker) | 0 = no; 1 = yes | 6 | 0.038257304 | Standard |
| Insurance status - Private Only (privatepublicins_1.0) | 0 = uninsured; 1 = private only; 2 = public only;  3 = private & public | 7 | 0.033338277 | Socioeconomic |
| Insurance status - Private & Public (privatepublicins_3.0) | 0 = uninsured; 1 = private only; 2 = public only;  3 = private & public | 8 | 0.027698644 | Socioeconomic |
| Insured (insured) | 0 = no; 1 = yes | 9 | 0.027554328 | Socioeconomic |
| Insurance status - Uninsured (privatepublicins_0.0) | 0 = uninsured; 1 = private only; 2 = public only;  3 = private & public | 10 | 0.025821705 | Socioeconomic |
| HDL cholesterol (hdl) | Fasting HDL cholesterol level (mg/dL) | 11 | 0.022134201 | Standard |
| Waist (waist) | Waist circumference (cm) | 12 | 0.02156781 | Standard |
| Discrimination burden (discrmburden) | Higher scores = greater lifetime burden from discrimination | 13 | 0.0205862 | Psychosocial |
| 1/2 mile simple favorable food stores (s0fav) | Higher scores = more favorable food stores available within area to resident | 14 | 0.020243589 | Environmental |
| 1 mile simple modified retail food environment index exclude alcohol (s1mrfei_noalc) | Higher scores = greater proportion healthy food retailers available within area to resident | 15 | 0.018105166 | Environmental |
| Percent of 1/2 mile buffer in land use data (pctlu0) | Higher % = greater proportion of buffer area in land use data | 16 | 0.016688451 | Environmental |
| Family income (fmlyinc) | 1 = poor; 2 = lower-middle; 3 = upper-middle; 4 = affluent | 17 | 0.015775063 | Socioeconomic |
| Global Stress Score (perceivedstress) | Higher scores = higher levels of stress in past 12 months | 18 | 0.014333366 | Psychosocial |
| 1 mile kernel modified retail food environment index exclude alcohol (k1mrfei_noalc) | Higher scores = greater proportion healthy food retailers available within area to resident | 19 | 0.014159857 | Environmental |
| Percent of 1 mile buffer in land use data (pctlu1) | Higher % = greater proportion of buffer area in land use data | 20 | 0.013936951 | Environmental |
| Ankle brachial index (abi) | Ratio of systolic blood pressure (SBP) measured at the ankle to SBP measures at the arm | 21 | 0.013311411 | Standard |
| 1 mile kernel modified retail food environment index include alcohol (k1mrfei_tot) | Higher scores = greater proportion healthy food retailers available within area to resident | 22 | 0.01302314 | Environmental |
| 1 mile simple modified retail food environment index include alcohol (s1mrfei_tot) | Higher scores = greater proportion healthy food retailers available within area to resident | 23 | 0.012695896 | Environmental |
| Employment Status (Not Employed) (occupation_not_employed) | 1 = management or professional; 2 = service; 3 = sales; 4 = farming; 5 = construction; 6 = production; 7 = military; 8 = sick; 9 = unemployed; 10 = homemaker; 11 = retired; 12 = student; 13 = other | 24 | 0.012643907 | Socioeconomic |
| % Household w/no vehicle (vehicle_none) | Proportion of households in census tract with no vehicle | 25 | 0.012138113 | Environmental |
| 1/2 mile kernel total physical activities + instructional + instructional + water (k0pai) | Higher scores = more activity resources available within area to resident | 26 | 0.011339255 | Environmental |
| Hemoglobin A1C (hba1c) | Average blood glucose level for the past 2-3 months (% of glycated hemoglobin) | 27 | 0.01104396 | Standard |
| 1/2 mile kernel modified retail food environment index exclude alcohol (k0mrfei_noalc) | Higher scores = greater proportion healthy food retailers available within area to resident | 28 | 0.01100445 | Environmental |
| 1/2 mile kernel outdoor total physical activities + instructional + water (k0opai) | Higher scores = more activity resources available within area to resident | 29 | 0.010811341 | Environmental |
| Percent of 1/4 mile buffer in land use data (pctlu14) | Higher % = greater proportion of buffer area in land use data | 30 | 0.010540697 | Environmental |
| Population density per sq mile 1 mile (popdenmi1) | Higher scores = greater population density in area | 31 | 0.010117516 | Environmental |
| 1/2 mile kernel indoor total physical activities + instructional (k0ipai) | Higher scores = more activity resources available within area to resident | 32 | 0.010052403 | Environmental |
| Insurance status - Public Only (privatepublicins_2.0) | 0 = uninsured; 1 = private only; 2 = public only;  3 = private & public | 33 | 0.009727942 | Socioeconomic |
| Systolic blood pressure (sbp) | Higher = greater average systolic blood pressure (mmHg) across two readings | 34 | 0.009007272 | Standard |
| % Household with interest, dividend, rental income (inc_intdivrent) | Proportion of households in census tract with income from interest, dividend, and/or rentals | 35 | 0.008577956 | Environmental |
| Percent Retail 1 mile (pret1) | Higher % = greater proportion of retail land use in buffer area | 36 | 0.008457593 | Environmental |
| 3 mile simple modified retail food environment index include alcohol (s3mrfei_tot) | Higher scores = greater proportion healthy food retailers available within area to resident | 37 | 0.008401899 | Environmental |
| 1/2 mile simple walking (s0walk) | Higher scores = more walking destinations available within area to resident | 38 | 0.00816442 | Environmental |
| Physical Activity (pa3cat) | 0 = poor health; 1 = intermediate health;  2 = ideal health | 39 | 0.008103816 | Standard |
| Population density per sq km 1 mile (popdenkm1) | Higher scores = greater population density in area | 40 | 0.008042654 | Environmental |
| % asian non-hispanic (race_asiannh) | Proportion of census tract identifying as Non-Hispanic Asian | 41 | 0.007658348 | Environmental |
| 1/2 mile simple modified retail food environment index exclude alcohol (s0mrfei_noalc) | Higher scores = greater proportion healthy food retailers available within area to resident | 42 | 0.007594044 | Environmental |
| Education - Less than high school Graduate (edu3cat_less_HSgrad) | 0 = less than high school; 1 = high school graduate or GED; 2 = attended vocational school, trade school, or college | 43 | 0.007451973 | Socioeconomic |
| Education - High school graduated (edu3cat_HSgrad) | 0 = less than high school; 1 = high school graduate or GED; 2 = attended vocational school, trade school, or college | 44 | 0.007225247 | Socioeconomic |
| 3 mile kernel modified retail food environment index include alcohol (k3mrfei_tot) | Higher scores = greater proportion healthy food retailers available within area to resident | 45 | 0.007172713 | Environmental |
| Depressive Symptoms Score (depression) | Higher scores = greater depressive symptoms in past week | 46 | 0.006927913 | Psychosocial |
| 1/2 mile simple total food stores (s0totfood) | Higher scores = more food stores available within area to resident | 47 | 0.006815455 | Environmental |
| Percent Residential 1 mile (pres1) | Higher % = greater proportion of residential land use in buffer area | 48 | 0.006477597 | Environmental |
| 1/2 mile simple social engagement (s0soc) | Higher scores = more social destinations available within area to resident | 49 | 0.006400034 | Environmental |
| LDL cholesterol (ldl) | Fasting LDL cholesterol level (mg/dL) | 50 | 0.006385364 | Standard |
| 1 mile simple outdoor total physical activities + instructional + water (s1opai) | Higher scores = more activity resources available within area to resident | 51 | 0.00636836 | Environmental |
| % in same house (samehouse) | Proportion of residents in census tract living in the same house as prior year | 52 | 0.006346437 | Environmental |
| 1 mile simple unfavorable food stores (s1unfav) | Higher scores = more unfavorable food stores available within area to resident | 53 | 0.006229683 | Environmental |
| % Household owner occupied (ownerocc_hh) | Proportion of owner-occupied households in census tract | 54 | 0.006178985 | Environmental |
| 1 mile kernel favorable food stores (k1fav) | Higher scores = more favorable food stores available within area to resident | 55 | 0.005936698 | Environmental |
| 1/2 mile kernel modified retail food environment index include alcohol (k0mrfei_tot) | Higher scores = greater proportion healthy food retailers available within area to resident | 56 | 0.005903071 | Environmental |
| 3 mile simple social engagement (s3soc) | Higher scores = more social destinations available within area to resident | 57 | 0.005496035 | Environmental |
| 1/2 mile simple modified retail food environment index include alcohol (s0mrfei_tot) | Higher scores = greater proportion healthy food retailers available within area to resident | 58 | 0.005436841 | Environmental |
| 3 mile simple unfavorable food stores excluding alcohol (s3unfavfo) | Higher scores = more unfavorable food stores available within area to resident | 59 | 0.005289028 | Environmental |
| % white non-hispanic (race_whitenh) | Proportion of census tract identifying as Non-Hispanic White | 60 | 0.005201358 | Environmental |
| Percent Commercial 1/4 mile (pcom14) | Higher % = greater proportion of commercial land use in buffer area | 61 | 0.00509766 | Environmental |
| 3 mile simple modified retail food environment index exclude alcohol (s3mrfei_noalc) | Higher scores = greater proportion healthy food retailers available within area to resident | 62 | 0.0050967 | Environmental |
| Median owner Household cost (hucost_medownval) | Higher = greater household owner cost in census tract | 63 | 0.005084705 | Environmental |
| Percent Residential 1/2 mile (pres0) | Higher % = greater proportion of residential land use in buffer area | 64 | 0.005026957 | Environmental |
| 3 mile simple favorable food stores (s3fav) | Higher scores = more favorable food stores available within area to resident | 65 | 0.004966949 | Environmental |
| Fasting glucose (fpg) | Fasting plasma glucose level (mg/dL) | 66 | 0.00496043 | Standard |
| % Household w/no telephone (phone_none) | Proportion of households in census tract with no telephone | 67 | 0.004716397 | Environmental |
| Age & gender adjusted Unconditional Empirical Bayes Estimate (UEBE) for NB Problem PCA-based (nppca_uebe) | Higher scores = greater loading on neighborhood problems scale (including excessive noise, heavy traffic or speeding cars, lack of access to adequate food and/or shopping, lack of parks and playground, trash and litter, no sidewalks and poorly maintained sidewalks) | 68 | 0.004698085 | Environmental |
| 1/2 mile kernel favorable food stores (k0fav) | Higher scores = more favorable food stores available within area to resident | 69 | 0.004563835 | Environmental |
| Network Ratio 1/2 mile (netratio0) | Higher = more connected street network within area | 70 | 0.004526236 | Environmental |
| Lifetime discrimination (lifetimediscrm) | Higher scores = greater number of lifetime major life event discrimination experiences | 71 | 0.004480505 | Psychosocial |
| 3 mile kernel modified retail food environment index exclude alcohol (k3mrfei_noalc) | Higher scores = greater proportion healthy food retailers available within area to resident | 72 | 0.004361858 | Environmental |
| 1 mile kernel total physical activities + instructional + instructional + water (k1pai) | Higher scores = more activity resources available within area to resident | 73 | 0.004271763 | Environmental |
| 3 mile kernel total food stores (k3totfood) | Higher scores = more food stores available within area to resident | 74 | 0.004050082 | Environmental |
| 1/2 mile kernel total stores (k0totstr) | Higher scores = more stores available within area to resident | 75 | 0.003885235 | Environmental |
| 3 mile kernel unfavorable food stores excluding alcohol (k3unfavfo) | Higher scores = more unfavorable food stores available within area to resident | 76 | 0.003851251 | Environmental |
| 3 mile kernel outdoor total physical activities + instructional + water (k3opai) | Higher scores = more activity resources available within area to resident | 77 | 0.003696775 | Environmental |
| Physical activity during leisure time (activeindex) | Higher scores = greater physical activity during leisure time | 78 | 0.003692017 | Standard |
| 3 mile kernel indoor total physical activities + instructional (k3ipai) | Higher scores = more activity resources available within area to resident | 79 | 0.003607113 | Environmental |
| 1 mile simple total food stores (s1totfood) | Higher scores = more food stores available within area to resident | 80 | 0.00348799 | Environmental |
| Number of intersections in 1/2 mile buffer (intcnt0) | Higher = more intersections within area | 81 | 0.003452139 | Environmental |
| 3 mile simple outdoor total physical activities + instructional + water (s3opai) | Higher scores = more activity resources available within area to resident | 82 | 0.003431595 | Environmental |
| % 25+ with minimum High School education (educ_minhs) | Proportion of individuals ages 25 and over in census tract with at least a high school education | 83 | 0.003387103 | Environmental |
| 1/2 mile simple total stores (s0totstr) | Higher scores = more stores available within area to resident | 84 | 0.003341125 | Environmental |
| % Household w/income >= $50,000 (inc_hhge50k) | Proportion of households in census tract with annual income equal to or greater than $50,000 | 85 | 0.003329823 | Environmental |
| 3 mile kernel total stores (k3totstr) | Higher scores = more stores available within area to resident | 86 | 0.003306813 | Environmental |
| % other non-Hispanic (race_othernh) | Proportion of census tract identifying as Other Non-Hispanic | 87 | 0.003228573 | Environmental |
| Total cholesterol (totchol) | Fasting total cholesterol (mg/dL) | 88 | 0.003171702 | Standard |
| 1 mile simple walking (s1walk) | Higher scores = more walking destinations available within area to resident | 89 | 0.003151486 | Environmental |
| Average number of drinks per week (alcw) | Average number of alcoholic drinks consumed by participant in a week | 90 | 0.003137378 | Standard |
| Employment Status (Employed) (occupation_employed) | 1 = management or professional; 2 = service; 3 = sales; 4 = farming; 5 = construction; 6 = production; 7 = military; 8 = sick; 9 = unemployed; 10 = homemaker; 11 = retired; 12 = student; 13 = other | 91 | 0.003063538 | Socioeconomic |
| 3 mile kernel favorable food stores (k3fav) | Higher scores = more favorable food stores available within area to resident | 92 | 0.003021204 | Environmental |
| Percent Commercial 1/2 mile (pcom0) | Higher % = greater proportion of commercial land use in buffer area | 93 | 0.003014129 | Environmental |
| Age & gender adjusted Unconditional Empirical Bayes Estimate (UEBE) for Violence PCA-based (vopca_uebe) | Higher scores = greater loading on violence scale (including frequency of fights with weapons, violent arguments between neighbors, gang fights, sexual assault or rape, robbery or mugging) | 94 | 0.002836526 | Environmental |
| Population density including land + water (per km square) (popden_tot) | Higher scores = greater population density in area | 95 | 0.002811543 | Environmental |
| 3 mile kernel social engagement (k3soc) | Higher scores = more social destinations available within area to resident | 96 | 0.002768095 | Environmental |
| % unemployed (unemployed) | Proportion of unemployed residents in census tract | 97 | 0.002759212 | Environmental |
| 1 mile kernel unfavorable food stores excluding alcohol (k1unfavfo) | Higher scores = more unfavorable food stores available within area to resident | 98 | 0.00272698 | Environmental |
| Number of intersections in 1 mile buffer (intcnt1) | Higher = more intersections within area | 99 | 0.002694568 | Environmental |
| Age & gender adjusted Unconditional Empirical Bayes Estimate (UEBE) for Social Cohesion PCA-based (scpca_uebe) | Higher scores = greater loading on social cohesion scale (including close knit neighborhood, willingness to help, getting along, trustworthiness, similar values, safety from crime) | 100 | 0.002637839 | Environmental |
| 1 mile kernel walking (k1walk) | Higher scores = more walking destinations available within area to resident | 101 | 0.002616555 | Environmental |
| % black non-hispanic (race_blacknh) | Proportion of census tract identifying as Non-Hispanic Black | 102 | 0.002534593 | Environmental |
| 1/2 mile kernel total food stores (k0totfood) | Higher scores = more food stores available within area to resident | 103 | 0.002524349 | Environmental |
| % 25+ with minimum bachelor degree (educ_minba) | Proportion of individuals ages 25 and over in census tract with at least a bachelor degree | 104 | 0.002503961 | Environmental |
| % managerial occupation (occup_i) | Proportion of residents in census tract with managerial occupation | 105 | 0.002458961 | Environmental |
| % below poverty (pov) | Proportion of residents in census tract living below the poverty line | 106 | 0.002425784 | Environmental |
| Network Ratio 1 mile (netratio1) | Higher = more connected street network within area | 107 | 0.002407137 | Environmental |
| 1/2 mile simple indoor total physical activities + instructional (s0ipai) | Higher scores = more activity resources available within area to resident | 108 | 0.002405046 | Environmental |
| Triglycerides (trigs) | Fasting triglyceride level (mg/dL) | 109 | 0.002329222 | Standard |
| Alcohol drinking in the past 12 months (alc) | 0 = yes; 1 = no | 110 | 0.002310867 | Standard |
| % not in labor force (notinlaborforce) | Proportion of residents in census tract who are not in the labor force | 111 | 0.002298128 | Environmental |
| 3 mile simple indoor total physical activities + instructional (s3ipai) | Higher scores = more activity resources available within area to resident | 112 | 0.002156022 | Environmental |
| % hispanic (race_hisp) | Proportion of census tract identifying as Hispanic | 113 | 0.002155989 | Environmental |
| 1 mile kernel outdoor total physical activities + instructional + water (k1opai) | Higher scores = more activity resources available within area to resident | 114 | 0.002136639 | Environmental |
| 3 mile simple total physical activities + instructional + water (s3pai) | Higher scores = more activity resources available within area to resident | 115 | 0.002118107 | Environmental |
| 1 mile simple total physical activities + instructional + water (s1pai) | Higher scores = more activity resources available within area to resident | 116 | 0.002112799 | Environmental |
| Network Ratio 1/4 mile (netratio14) | Higher = more connected street network within area | 117 | 0.002084885 | Environmental |
| Percent Retail 1/2 mile (pret0) | Higher % = greater proportion of retail land use in buffer area | 118 | 0.002071796 | Environmental |
| 1 mile simple favorable food stores (s1fav) | Higher scores = more favorable food stores available within area to resident | 119 | 0.002053868 | Environmental |
| Population density per sq mile 1/2 mile (popdenmi0) | Higher scores = greater population density in area | 120 | 0.002002164 | Environmental |
| Falls within county boundaries for land use data (inlucounty) | 0 = outside; 1 = within | 121 | 0.001867527 | Environmental |
| 1/2 mile kernel walking (k0walk) | Higher scores = more walking destinations available within area to resident | 122 | 0.001727319 | Environmental |
| 1 mile kernel total stores (k1totstr) | Higher scores = more stores available within area to resident | 123 | 0.00170326 | Environmental |
| 1/2 mile simple unfavorable food stores excluding alcohol (s0unfavfo) | Higher scores = more unfavorable food stores available within area to resident | 124 | 0.001609578 | Environmental |
| 1 mile simple indoor total physical activities + instructional (s1ipai) | Higher scores = more activity resources available within area to resident | 125 | 0.001591956 | Environmental |
| Number of intersections in 1/4 mile buffer (intcnt14) | Higher = more intersections within area | 126 | 0.001589094 | Environmental |
| 1 mile kernel unfavorable food stores (k1unfav) | Higher scores = more unfavorable food stores available within area to resident | 127 | 0.001588774 | Environmental |
| Percent Commercial 1 mile (pcom1) | Higher % = greater proportion of commercial land use in buffer area | 128 | 0.001558852 | Environmental |
| 1/2 mile simple unfavorable food stores (s0unfav) | Higher scores = more unfavorable food stores available within area to resident | 129 | 0.001395928 | Environmental |
| % Household with crowding > 1 person per room (crowd_gt1_ppr) | Proportion of households in census tract with crowding (more than 1 person per room) | 130 | 0.001193535 | Environmental |
| 1 mile kernel total food stores (k1totfood) | Higher scores = more food stores available within area to resident | 131 | 0.001170187 | Environmental |
| BMI (bmi) | Body mass index (kg/m^2^) | 132 | 0.001164621 | Standard |
| 1 mile kernel indoor total physical activities + instructional (k1ipai) | Higher scores = more activity resources available within area to resident | 133 | 0.001127374 | Environmental |
| 1/2 mile kernel unfavorable food stores excluding alcohol (k0unfavfo) | Higher scores = more unfavorable food stores available within area to resident | 134 | 0.001101998 | Environmental |
| Age (age) | Age of participant in years | 135 | 0.001010641 | Standard |
| Population density per sq km 1/4 mile (popdenkm14) | Higher scores = greater population density in area | 136 | 0.000942001 | Environmental |
| 1 mile simple total stores (s1totstr) | Higher scores = more stores available within area to resident | 137 | 0.00088051 | Environmental |
| 1/2 mile kernel unfavorable food stores (k0unfav) | Higher scores = more unfavorable food stores available within area to resident | 138 | 0.000860752 | Environmental |
| 3 mile simple total food stores (s3totfood) | Higher scores = more food stores available within area to resident | 139 | 0.000768907 | Environmental |
| Population density per sq mile 1/4 mile (popdenmi14) | Higher scores = greater population density in area | 140 | 0.000765928 | Environmental |
| 3 mile kernel walking (k3walk) | Higher scores = more walking destinations available within area to resident | 141 | 0.000711171 | Environmental |
| 1/2 mile simple total physical activities + instructional + water (s0pai) | Higher scores = more activity resources available within area to resident | 142 | 0.000684105 | Environmental |
| 3 mile simple total stores (s3totstr) | Higher scores = more stores available within area to resident | 143 | 0.000660525 | Environmental |
| Population density per sq km 1/2 mile (popdenkm0) | Higher scores = greater population density in area | 144 | 0.000602762 | Environmental |
| % Household occupied (hu_sampleocc) | Proportion of occupied households in census tract | 145 | 0.000571933 | Environmental |
| 1 mile simple social engagement (s1soc) | Higher scores = more social destinations available within area to resident | 146 | 0.000397867 | Environmental |
| 1 mile kernel social engagement (k1soc) | Higher scores = more social destinations available within area to resident | 147 | 0.000374736 | Environmental |
| 3 mile kernel total physical activities + instructional +water (k3pai) | Higher scores = more activity resources available within area to resident | 148 | 0.000365067 | Environmental |
| 3 mile kernel unfavorable food stores (k3unfav) | Higher scores = more unfavorable food stores available within area to resident | 149 | 0.000345417 | Environmental |
| Diastolic blood pressure (dbp) | Higher = greater average diastolic blood pressure (mmHg) across two readings | 150 | 0.000293316 | Standard |
| % foreign born (birth_foreign) | Proportion of residents in census tract who are foreign born | 151 | 0.000287492 | Environmental |
| Weekly stress score (weeklystress) | Higher scores = more minor stressful events in past week | 152 | 0.000286142 | Psychosocial |
| 1/2 mile kernel social engagement (k0soc) | Higher scores = more social destinations available within area to resident | 153 | 0.000215853 | Environmental |
| 3 mile simple unfavorable food stores (s3unfav) | Higher scores = more unfavorable food stores available within area to resident | 154 | 0.000160466 | Environmental |
| Percent Retail 1/4 mile (pret14) | Higher % = greater proportion of retail land use in buffer area | 155 | 0.000141868 | Environmental |
| Percent Residential 1/4 mile (pres14) | Higher % = greater proportion of residential land use in buffer area | 156 | 0.000137821 | Environmental |
| 3 mile simple walking (s3walk) | Higher scores = more walking destinations available within area to resident | 157 | 0.00012971 | Environmental |
| % with public assistance (inc_pubass) | Proportion of residents in census tract with public assistance | 158 | 9.97E-05 | Environmental |
| 1 mile simple unfavorable food stores excluding alcohol (s1unfavfo) | Higher scores = more unfavorable food stores available within area to resident | 159 | 9.35E-05 | Environmental |
| Daily discrimination (dailydiscr) | Higher scores = more day-to-day experiences of unfair treatment | 160 | 8.53E-05 | Psychosocial |
| Median Household income (inc_medhh) | Higher = greater household income in census tract | 161 | 1.67E-07 | Environmental |
